# The convivial and the pastoral in patient–doctor relationships: a multi‐country study of patient stories of care, choice and medical authority in cancer diagnostic processes

**DOI:** 10.1111/1467-9566.13067

**Published:** 2020-02-26

**Authors:** John I. MacArtney, Rikke S. Andersen, Marlene Malmström, Birgit Rasmussen, Sue Ziebland

**Affiliations:** ^1^ Unit of Academic Primary Care Warwick Medical School University of Warwick Warwick UK; ^2^ Research Centre for Cancer Diagnosis in Primary Care Research Unit of General Practice & Department of Anthropology Aarhus University Aarhus Denmark; ^3^ The Institute for Palliative Care Lund University and Region Skåne Lund Sweden; ^4^ Lund University Department of Health Sciences Lund Sweden; ^5^ Health Experiences Research Group Nuffield Department of Primary Care Health Sciences University of Oxford Oxford UK

**Keywords:** Patient‐doctor relationship, cancer experiences, care, choice, medical authority, international comparison

## Abstract

Experiences of cancer diagnosis are changing in light of both the increasingly technological‐clinical diagnostic processes and the socio‐political context in which interpersonal relations take place. This has raised questions about how we might understand patient–doctor relationship marked by asymmetries of knowledge and social capital, but that emphasise patients’ empowered choices and individualised care. As part of an interview study of 155 participants with bowel or lung cancer across Denmark, England and Sweden, we explored participants’ stories of the decisions made during their cancer diagnostic process. By focusing on the intersections of care, choice and medical authority – a convivial pastoral dynamic – we provide a conceptual analysis of the normative ambivalences in people's stories of their cancer diagnosis. We found that participants drew from care, choice and medical authority to emphasise their relationality and interdependence with their doctors in their stories of their diagnosis. Importantly negotiations of an asymmetrical patient–doctor relationship were part of an on‐going realisation of the healthcare processes as a human endeavour. We were therefore able to draw attention to the limitations of dichotomising emancipatory‐empowerment discourses and argue for a theorisation of the patient–doctor relationship as a contextually bounded and relationally ambivalent humanity.

## Introduction

A cancer diagnosis can be both a single moment of categorisation and a process through which a doctor and patient collaborate their respective expertise (Jutel and Dew 2014). Decisions are located within the lived context within which illness takes place (Andersen and Risør 2014) and draw on moral and cultural preferences for treatment (Lock and Kaufert 2001). Individual experiences of diagnosis can therefore be particular to a given national health system, but also draw on systems and structures of power and knowledge that are recognisable across countries (Brown *et al*. 2014). Across Europe (and worldwide) there has been a shared endeavour in cancer care to improve patient survival and experiences of diagnosis, from biomedical efforts to reduce diagnostic ambivalence to an intense policy focus on patient‐centred care, shared decision‐making and patient empowerment (Brown *et al*. 2014, Kerr *et al*. 2018, Rubin *et al*. 2015, Ziebland *et al*. 2018). After several decades of intensive research and intervention survival rates are broadly improving (Holleczek *et al*. 2015, Walters *et al*. 2015). However, those interventions have also served to increase the complexity of embodied knowledge and practices around cancer diagnostic processes (Andersen 2017, Kerr *et al*. 2018, Ziebland *et al*. 2018). This has come at a time when greater responsibility has been argued to lie with the patient for their health and care decisions, not just normatively (Buetow and Elwyn 2006), but legally (Chan *et al*. 2017).

It is therefore perhaps not surprising that the patent–doctor relationship itself is seen to be opening its next chapter within which new relations of care are being formed (Buetow *et al*. 2009, Fotaki 2011, Kirkpatrick *et al*. 2009, Pilnick *et al*. 2009). To better understand this, we draw on our qualitative research study of lung and colorectal cancer patients’ experiences of their pathways to diagnosis in Denmark, England and Sweden. In contrast to earlier publications from this study (Andersen *et al*. 2019, MacArtney *et al*. 2017, Malmström *et al*. 2018, Ziebland *et al*. 2018) that drew on cross country comparisons to highlight opportunities for service improvement, this article provides a sociological analysis of care, choice and medical authority in patients’ stories of their cancer diagnosis to further our understanding of the changing patient–doctor relationship. To help us do this we draw on two conceptualisations of asymmetrical relations in late‐modern society – the convivial and the pastoral (Gilroy 2004, Rose 2007). By doing so we aim to provide a theoretically informed exploration of the normative ambivalences that arise when care, choice and medical authority intersect within the patient–doctor relationship.

### *Care, choice and medical authority in patient*–*doctor relations*

Explorations of care, choice and medical authority have investigated what is at stake in the production of kinship and citizenship, as well as how state‐driven and institutional transitions in care and choice alter existing notions of relatedness, including in the patent–doctor relationship (Mol 2008, Rose 2007, Wilkinson and Kleinman 2016). What has become particularly evident are the ways that experiences and discourses of care, choice and medical authority are interrelated in people's experiences of healthcare.

A conceptual intersectionality is particularly notable in the ways that care and choice have been utilised as part of a patient empowerment or ‘service user emancipation’ strategy (Bevir *et al*. 2019: 1), which holds considerable normative sway in health policy via calls for patient‐centred, shared or co‐produced care. Both within and beyond healthcare policy sociologists have found heterogeneous understandings and practices of care and choice. Care has been argued to provide an alternative understanding of health and illness beyond medicalised concerns with the diseased body, which can help attune doctors to patients’ holistic needs (Mol 2008). Indeed, care has become the focus of intense political concern, professional consideration and evidence‐based scrutiny in a broad range of healthcare arenas (e.g. NHS Constitution; Patient Recorded Outcome Measures (PROMS); and, Quality and Outcome Frameworks (QOFs)). As a consquence, care has become knowable through the material administration of the clinical process, structuring healthcare from patient–doctor interactions to the management and expectations of good governance (Andersen and Vedsted 2015). What care is and how it is understood therefore stretches from ‘scientific‐bureaucratic medicine’ (Harrison and Wood 2000: 26) to the more humanistic and emancipatory ideals that have been associated with it (e.g. Gilligan 1993, Mol 2008).

Similarly choice is understood as an important discourse through which good care has come to be valued (Latimer *et al*. 2017, Mol 2008). Importantly, understandings of choice often draw on multiple assumptions that can confound attempts to define what is a good patient–doctor relationship. What is a good choice might differ depending upon whether it is understood as a choice promoting ideas of market efficiency in the context of competition (Davies 2017), a choice as a practice of liberty in a democratic process (Rose 2017) or a choice of an autonomous rational person (Latimer *et al*. 2017). As well as patient empowerment initiatives, policies and movements, ideas of care and choice have helped inform the ethos behind training clinicians with improved and efficacious interpersonal and communications skills (Elwyn *et al*. 2017). For both the patient and their doctor this milieu of care and choice forms part of a broad discursive and normative repertoire from which they can both draw within the patient–doctor relationship.

Intersections of care and choice have also affected how medical authority is to be understood and have contributed to a new normative context of ambivalent power relations. Attempts have been made to (re)locate the locus of medical authority, such as framing the relationship as one of ‘constrained collaboration’ (Vinson 2016), or ‘selective paternalism’ (Drolet and White 2012). Others have questioned whether the emphasis on patient autonomy and decision‐making in discourses of care and choice is part of a role convergence (Buetow *et al*. 2009), and described how the administration of care, choice and medical authority is part of the de‐professionalisation of medical care (Shore and Wright 2015). What such findings illuminate are assumptions about the patient–doctor relationship that draw on Schmittian winner–loser dichotomies (e.g. active v passive; mind v body; thinking v action) with associated normative implications of what is right and wrong (Davies 2016). Understood like this, a patient in the cancer diagnostic process who invokes their subjective, embodied and cultural choices finds their preferences contextualised in such a way that they are open to being – epistemologically and morally – subsumed within medicine's evidence‐based diagnostic ambivalence. Analysis of patient experiences of care, choice and medical authority are then only understandable as being *limited* to acts of concordance, through restricted options and/or non‐choices (Charles *et al*. 1998, Ziebland *et al*. 2006). As a consequence of these assumed dichotomies, hierarchies of knowledge and the question of dominance resurface as problems that are better addressed within the patient–doctor relationship (Clinch and Benson 2013). Recommendations then follow that attend to these dichotomised understandings as part of a wider ‘neo‐communitarian’ behavioural policy strategy (Davies 2017: 34) for example, to train doctors’ in better communication skills or educate and empower patients (or both) (Bevir, Needham, and Waring, 2019).

In contrast, other reflections upon care, choice and medical authority have informed models of patient–doctor relationship that move away from framing interactions as one of a struggle for dominance, and instead emphasise the co‐construction of the relationship (Dzeng 2019, Pilnick and Dingwall 2011) or (re‐emphasised) the social and fiduciary obligations to each other (Parsons 1951, Williams 2005). Here patient and doctor roles and values are seen to be continually shifting, as is the socio‐political context within which patients, doctors and sociologists might assess any concordance in that relationship. Questions therefore remain about how we might understand patient–doctor relationship marked by asymmetries of knowledge and social capital, but that emphasise patients’ empowered choices and individualised care. What is needed is an exploration of these new ambivalences within healthcare that do not resort to the divisive or binary formations of authority, and that are better able to situate the normative implications of discourses of care and choice (Mol 2008).

### *Diagnosing cancer: A convivial pastoral ambivalence*

To better understand patients’ accounts of the patient–doctor relationship in the cancer diagnostic process we turned to two conceptualisations of power relations that have provided insight into the ambivalence of asymmetrical relations in late‐modern societies: pastoral power and conviviality.^1^

Drawing on Michel Foucault's (1982) earlier work, Nikolas Rose's (2007) conceptualisation of the new pastoral power relations describes a form of collectivising and individualising power that are taking place in a plural and contested field such as cancer diagnosis. These fields are traversed by professional codes, empirical findings, attitudes and criteria of third‐party users, the advice of self‐help organisations, and the critical reflections of many groups. Characterised by the advocating of ethical principles of informed consent, autonomy, voluntary action, choice, responsibility and non‐direction, this form of pastoral power is therefore best characterised as ‘relational’ (Rose 2007: 74) as it entails an analysis of the dynamic and negotiated interaction between the individual (patient) and the ‘pastor’ (doctor). Although sometimes taken as a form or extension of governmentality theory, we prefer to utilise pastoral power to, ‘shift attention to the question of how discourses translate into subjectivity, action and material consequence – and to the active role of agents (pastors and others) in this process’ (Martin and Waring 2018: 8). However, while pastoral power helps draw attention to the importance of the ‘bidirectional affective entanglements’ in the systems and structures of care, choice and medical authority (Rose 2007: 74), we also need to be sensitised to ways of understanding the lived asymmetrical interpersonal relations that participants’ accounts might describe.

To do this we draw on the concept of conviviality, which has been used to describe forms of personal and creative interdependence between people, their use of ‘tools’, and their environment (Illich 2009 [1973]). In explorations of unequal relations in multiculture Gilroy (2004) has used conviviality to highlight some of the ways that structural, systemic and cultural differences are lived in congenial, reciprocal, friendly and trusting ways to produce new expressive and affective relations (Nayak 2017). Conviviality attends to the ambivalences in the everydayness of how people live together. Drawing on the Spanish idea of *convivencia* it is more than mere ‘happy togetherness’, as it includes the frictions and conflicts of negotiating a shared outcome (Wise and Noble 2016). Going beyond an ontological observation or ethical imperative, it can also be understood as a ‘convivial epistemology’ that provides the necessary sociocultural theoretical sensitisation to ‘mediating instrumentalities’, which add layers of with‐ness allowing people to understand things as they ‘are’ (Boisvert 2010: 63). As such we must attend to the performative, situated and temporal dimensions of convivial discourses to explore their ways they mediate (open up, include, bring together or close down, exclude, separate) (Wise and Noble 2016). Applied to participants’ stories of their cancer diagnosis, we suggest an analysis focusing on the convivial pastoral ambivalence to provide an opportunity to connect the systemic and structural context of health care to their experiences of the communicative and interpersonal practices of medical authority. In what follows we therefore seek to provide a conceptualisation of the patient–doctor relationship that – like the socio‐political and biomedical discourses that it draws on – transcends national borders, but that is also sensitive to the local challenges posed by the changes to care, choice and medical authority.

## Methods

### Recruitment

Approvals for research ethics and governance were obtained separately according to the requirements in each country.^2^ Purposive sampling (Coyne 1997) within each country was used to seek people within 6 months of diagnosis of lung or bowel cancer and to achieve gender balance. Variation was sought across age, urban and rural locations, pathways to diagnosis, and (where possible) self‐reported stage at diagnosis. Recruitment and interviewing took place concurrently in the three countries throughout 2015. A sociologist in England and an anthropologist in Denmark recruited and conducted all the interviews in their countries, while three nurse researchers trained in qualitative research methods collaborated in different parts of Sweden. A total of 155 interviews were conducted (see Table 1).

**Table 1:** Demographic characteristics across the three countries

	Denmark		England		Sweden	
	CC*	LC**	CC	LC	CC	LC
Number of participants	28	22	25	20	30	30
Percentage female	46%	36%	48%	50%	47%	50%
Age range						
31–50	2	0	4	*2*	2	2
51–70	19	15	13	12	14	21
71–90	7	7	8	6	14	7

*Colorectal cancer.

**Lung cancer.

Interviews were conducted in participants’ homes, unless they preferred another location. Interviews began with an open‐ended question: ‘Could you start by telling me, in your own words and in as much detail as you want, about everything that has happened since you first started to suspect there might be a problem with your health?’ The researcher then used a semi‐structured topic guide based on social science theories and cancer research literature about delays in presentation of symptoms and system delays in response (e.g. Andersen *et al*. 2010, Forbes *et al*. 2013, Locock *et al*. 2016, Ziebland *et al*. 2015). The research team had extensive discussions about the rationale and meaning of the interview items to ensure comparable data were collected. Patient and public involvement representatives with personal experience of lung or bowel cancer also commented on the topic guide (translated) in each country. Interviews were not reduced to discussing specific topics, but looked to open‐up reflections including those relevant to this paper, such as how they felt about key decision‐making periods of their care. This included exploring the first sensations or symptoms; how, why and when they sought help from a healthcare professional; what happened in those consultations and the investigations that followed; and, their reflections and thoughts about their experiences of the cancer diagnostic process. In particular we sought reflections on both what was said in clinical encounters and what they thought and felt about what was happening. The interviews lasted between 45 minutes to 1.5 hours and were audio recorded before being transcribed verbatim in the language of the interview.

### Analysis

Monthly teleconferences with the field research team (all of whom had a high level of spoken and written English) were held throughout the recruitment, data collection and analysis phases. Interview accounts were analysed for narrative themes that structured participant experiences. Data saturation was judged to have been reached in the analytic categories for all three countries before recruitment closed (Guest *et al*. 2006). To achieve this, in each country interview transcripts were imported into NVivo 10 for organising textual data. Each country conducted a separate thematic analysis using a coding frame developed through discussion in the teleconferences and based on the (anticipated) themes from the topic guide and emergent themes, which could be country specific (Pope *et al*. 2000). One 2‐day and one 4‐day analysis and writing workshops were held with the research teams from all three countries, along with regular teleconferences held throughout the study (see Chapple and Ziebland 2017). Emerging analyses were iteratively tested with each country's dataset in on‐going dialogue with the international team, drawing on existing knowledge and theoretical insights in the field. Swedish and Danish interview extracts were translated by the bi‐lingual researchers.

The methodology for this analysis is located within interpretive traditions in sociology (Silverman 2011). Specifically, our approach loops between conceptual and the lived experiences, with the aim to use each to better elucidate the other (Hacking 1995, MacArtney 2017). The analysis for this paper therefore draws on social theories to explore common features of cancer diagnosis across three countries with national healthcare systems. While differences between the three countries have been found (see MacArtney *et al*. 2017), the focus here was to provide insight into shared social and political norms of care and choice in participants’ stories of their diagnosis. To do this we approached the stories participants told us about their diagnosis – specifically the key moments and processes they identified – as living, local and specific (Harrington 2008). Our analysis of these stories then sought to identify possible normative connections between micro and macro contexts within which participants’ telling and our interpretation might take place (Andersen and Risør 2014). By doing so we were then able to provide an analysis of care, choice and medical authority and, in the Discussion, provide a conceptual contribution that draws on the convivial and the pastoral to understand better the patient–doctor relationship in cancer diagnosis.

## Findings

Our analysis of participant stories of their cancer diagnosis encapsulates a period that (for many, but not all) started with initial sensations of something being wrong, through initial consultation and various ensuing investigations, to the (post‐surgery) histological test results that identified and allocated the cancer within a clinical or genetic typology. During this diagnostic process patients can draw on cues and clues so that diagnosis is foreknown or becomes crystallised at a particular moment (Locock *et al*. 2016). As we analysed and reflected upon the interviews across the three countries we found that participants drew on understandings of care, choice and medical authority in their encounters with doctors in family (GP) practices, Emergency Rooms or oncology departments. In our findings we found that the intersections of care, choice and medical authority made participants’ stories of their diagnosis intelligible in two main ways. The first emphasises the relationality and interdependence of patients with their doctors in negotiations of the ambivalent space that intersections of care, choice and medical authority creates. So that what is too much, or too little, care or choice or medical authority is contextually bound and was expected to be individualised. The second sensitises us to the assumption in participant stories that these negotiations take place within an asymmetrical relationship, but one that is understood as part of an on‐going realisation of the healthcare processes as a human endeavour. That is, to be ‘cared for’ is no longer just assumed to be part of the patient–doctor relationship, but is an outcome to be achieved through a shared negotiation.

### Negotiating relational ambivalences in cancer diagnosis

During the diagnostic process many participants emphasised how the quality of their interactions with their doctors reflected expectations of being treated as an individual, and that this had to be done in the right way for them. Participants wanted their account of their sensations to be respected and taken seriously, sometimes asserting an expectation that they are experts of their bodies. Yet many participants also recognised the doctor's role to interpret those subjective sensations and make them intelligible within the diagnostic process. For some participants their doctors got this largely right and their stories of these moments can be seen to be indifferent to the asymmetries, containing little contestation or negotiation. But as we also explore, it is in those where the balance was not suitably individualised we can better see the contours of this ambivalence.

Improving interactions between patients and doctors has been an important goal for initiatives addressing care, choice and medical authority. Within the cancer diagnostic process the juxtaposition of these discourses brought a normatively ambivalent experience that needed to be worked out within the patient–doctor relationship. For example,

I think once you put yourself into the hands of the experts, let them get on with it and follow their advice. [er] That's what I have [erm] learned all my life and certainly in medical terms [erm] it, it seems absolutely clear to me that [erm] they know what they are doing… And they are at pains to [er] at the end of each discussion so when “Have you got any questions at all?” They are at pains to have their brains picked, so there was absolutely, there was no problem whatever about not being kept informed. At no point did I feel I was being somehow not taken notice of. People were very concerned to make sure that I knew exactly what was going on at every stage.

The participant effusively describes how the clinicians were ‘at pains to have their brains picked’. That is, time was provided for questions to be asked and answers explained to his satisfaction. What is noticeable is that he describes this not as the gathering of information upon which to base his empowered choice(s), but as part of a more receptive process of ‘being kept informed’. Importantly, as we return to below, this was done in such a way that he was able to consider himself as an active agent in the process who felt he was being ‘taken notice of’.

As the above excerpt also shows, there is an expectation that doctors open up the participant's capacity to interpret and understand what was happening during the diagnostic process, in a way that made sense and was appropriate to them. Some, like the following participant, had to take a more active role to ensure they got the information in a way that worked best for them.

P Yes. The [doctors] tried to explain and I even got them to speak a language that is not only medical language, with its strange words, but talk so you could understand.

I What did you do to get them to talk like that?

P Honestly, I said, “what you're talking about now, it's totally Greek to me. So if you do not talk in a language an ordinary person can understand then there is no point in you saying anything.”

I You said that? That's funny!

P I said, probably to most of the doctors when they spoke, “you have said ‘yadda‐yadda‐yadda’…” Then I said. “This is wasted time. There is no reason for you to sit and waste our time when you are talking to me in [medical] lingo”

The participant explains that she expects to be addressed in a way that opens‐up understanding and allow her to partake more fully in the interaction. The contestation described in the participant's story is not framed as a desire to usurp or replace the doctor or their medical authority, to which she appears somewhat indifferent. Rather, the participant wishes to be engaged in the conversation in a way she can understand. It is a communicative – rather than epistemic – equity that is prioritised and for this to occur the doctors need to express themselves in such a way that the participant can engage and respond to the information that is provided.

However, a fully involved conversation about the diagnosis was not always possible or necessary. It may seem self‐evident, but it was notable that for a number of participants the cultural context of a cancer diagnosis – in particular its life‐threatening seriousness and need for urgent treatment – affected how they understood the diagnostic process and evaluated the actions of the doctor's involved. Some participants reflected an indifference to discussing their diagnosis or treatment plans once the doctor had interpreted the clinical investigations as ‘cancer’. Other participants talked about how they were given the information ‘straight’ and how this similarly invoked equivocal reflections about their opportunities to discuss or reflect on the diagnosis with their doctor. Or as one participant put it, ‘you've got an opinion [but] you don't have an option’. That is, for these participants it was not always necessary to feel that they were making fully informed, empowered choices.

In other contexts, however, the expectation that doctors collaborate with patients meant that this ‘giving it straight’ approach to communication could be problematic; for example, when a doctor failed to take into account a patient's concerns. As with the following participant, who had been told that there was a ‘shadow’ on his lung when having an X‐ray for a broken back.

P But yeah, I mean, the, the nurses do a better job [of communicating] than the, the doctor's really. Because they know how, how you feel. A doctor, you're just a body, aren't you.

I Right. Is that how you felt with them?

P Well, yeah. They want to get the cancer out of you. So, you're just, ‘we need to get that out’, no matter what else has gone on. They need to stop and listen to people's fears.

When the participant was asked what those fears were, he explained that he was worried that he might not survive a further operation, having had major back surgery weeks before. He said, ‘I was angry that nobody was listening to me saying, “Will my body take it? And will my back cope with it?”’ Yet despite his significant worries about the prospect of having further surgery, the participant went on to reflect, ‘I know my body. But I also understood that they needed to get it done fast in case it spread’. What this participant's story of his anxieties of not being heard by his doctors allows us to highlight are the normative ambivalences that arise in his experiences of poor care and choice. That is, his evaluation of this moment in his diagnostic process is not reducible to an understanding of quality of communication or empowered choices. This is because his reflections on the poor care and choices can be contextualised within a further concern, such as this participant's understanding that a cancer diagnosis necessitates quick action that doctors are best placed to interpret and address. The participant would have appreciated better explanations from his doctors of what was happening and why, but his story helps illuminate how care, choice and medical authority intersect in participants’ sense making of their cancer diagnostic experiences.

Recognising the intersectionality of care, choice and medical authority in participant stories of their cancer diagnosis also helps to illuminate how some participants understood the importance of their doctor in individualising and contextualising the decisions they faced. As this participant explains,

The surgeon was a lot more matter‐of‐fact and not a chatty person. So you know, he just wants to get on with telling you what he can do and you take it or leave it, that was his, that was very much his manner, you know. ‘This is what I can offer, but I'm not forcing you down that line’ or you know, whereas in some ways I think it would have been a lot easier like in the olden days where they said, ‘Oh, now you're having an operation’ and you don't realise you've got any choice in the matter. But now, do I or don't I, you know, it's all, the balls in your court. You want to make the best decision for you. And I do feel that quality is what I want more than quantity. But it depends on the quality, you know, maybe you can't have like gold star quality but silver and silver might be better than nothing [laughs].

The surgeon provides the participant with all the information she needs about her (clinical) diagnosis and treatment options, but distances himself both interpersonally – ‘not a chatty person’ – and from directing her about what to do. The participant reflects that this puts her in a difficult position as she wants ‘to make the best decision’. She considers that the ‘quality’ of the information she has been given is ‘gold star’, but wonders whether ‘silver star’ ‘might be better than nothing’. Following on from her reflection about on the benefits of ‘the olden days’ where decisions are made for the patient, we interpret this as raising the question about the balance between being informed about decisions and having to make those decisions on your own. The participant's reflections suggest the surgeon had not found the right balance of care, choice and medical authority in this situation. That is, while avoiding the removal of ‘any choice’ there remained an expectation that the surgeon could direct the participant in the interpretation and individualisation of the information she had given to ensure the participant got the quality of care hoped for.

The doctor was therefore not just expected to provide information so that the patient can make choices, but to take part in the opening‐up of interpretation and understanding. As the following participant reflects, ‘I know what the diagnosis meant. I had read some articles’. However, simply having information about her diagnosis was not enough. When asked what support she was offered she remarked, ‘Well, basically they throw brochures in your face, don't they?’ Her partner clarified the problem, ‘Brochures are good if you have the resources to deal with it… but not if you do not’. And the participant went on to reflect,

… it is like being in a terrible accident. It would have been nice to have ‘a nanny’ to tell us about it, yes. To have a discussion with us that we did not get to have with the doctors. That was more like, they let us know [about the cancer], and that is how it is, and now you may leave. We did not have that brainstorming… It was very reductionistic.

The participant reflected that she knew what the diagnosis ‘meant’ from reading articles and the brochures, but she found her understanding was in some way restricted or lacking. What she sought was a ‘nanny’ who could first ‘tell us’ about the wider meaning and consequences of her diagnosis and then share a dialogue with her about that. Intersections of care, choice and medical authority extended beyond imparting information or good communication skills. They also anticipated some collaboration in the interpretation of what things mean for the participant and their life. Below we explore how we can better understand individualised and contextually bounded negotiations as part of an expectation whereby doctors are still expected to ‘nanny’ patients.

### The continuing importance of being cared ‘for’

In this section we explore some of the participants’ stories that focused on more doctor‐led decision‐making. We do this to consider how the asymmetry within the patient–doctor relationship is challenged, maintained or transformed in an increasingly complex diagnostic healthcare context that emphasises patient choice and responsibility that are to be negotiated via practices of shared decision‐making. We found these tensions throughout people's stories of diagnosis. For example,

I went to my family doctor and talked with her. So we went through different things and she listened to my lungs and things like that, and the heart and the whole thing. And so we came to the conclusion that we would do a chest X‐ray, for some reason we still do not understand why.

The participant consulted her family doctor and provided a somewhat collaborative description to the investigations undertaken. These discursive practices of collaboration suggest a mechanism through which the participant felt involved, even without understanding the GP's reasoning. In the rest of this subsection we will further consider these ambivalent, rather than determinative, spaces that discourses of care, choice and medical authority open‐up and how this affects patients’ normative expectations of patient–doctor relations in cancer diagnosis. In doing so we explore how these different – sometimes conflicting – ideas about healthcare might come together to suggest potentially novel ways for understanding the patient–doctor relationship. In particular, we explore how and when participants can appear indifferent to the asymmetries of knowledge and influence in the patient–doctor relationship.

In the following example the participant explained that he went to see his GP as he had blood in his stools. At the appointment he was told that he would ‘Skip all the intermediate stuff’ and be sent ‘hotline… straight to the hospital… for an endoscopy’. He summarised the GP's actions as,

… a fabulous decision, if you think of it. And, and I was a little bit disheartened because the way she did it was, she said, “There are… several reasons why you could see blood in your stools”. Obviously, meant since some of them are quite benign, but one of them is cancer – it was bowel cancer – and of course, that sent a shiver down my spine…”

The participant's description places an emphasis on the GP's decision to remove possible options, such as a stool sample, and send him straight for an endoscopy. The participant expressive reflection was that it was ‘a fabulous decision’. But we should also note that this is immediately qualified by ‘if you think of it’. The ambivalence invoked is contextualised in what follows: the participant draws on his own knowledge that ‘obviously’ there could be benign reasons for there being blood in his stools, but one possibility was cancer. This, he goes on to say, was a ‘disheartening’ prospect, an emotion embodied in a ‘shiver down [his] spine’. Furthermore, in recounting that there were other possible options available the participant acknowledges the possibility of choosing between alternative courses of action. Rather than querying his own lack of influence in that decision‐making process, the participant's story highlights the GP's concern for his wellbeing, suggesting a recognition of – and potential indifference to – the asymmetry in the decision‐making process.

The following story is particularly instructive in understanding asymmetrical relations in the patient–doctor relationship. The participant was a hospital doctor, whose story started with a description of the difficulty that she had getting a referral for further investigation from her GP. She had seen the GP twice, who had suggested the bleeding she was experiencing was due to constipation. Frustrated, and facing a third appointment with her GP, the participant spoke to a surgical colleague when at work, who was able to help her,

So then he takes ‐ exactly what one loves ‐ he takes over… So he took me and we went straight out to the nurse who you make appointments with, and so I got the [laboratory] referrals and so I got the appointments – everything I needed.

How might we understand the participant's wish to be cared for in the wider context where a doctor‐as‐patient might be argued to be close to being the idealised autonomous patient who is an empowered expert of her body? We suggest that this story allows for an interpretation other than being an ideal type of an emancipated–empowered patient. Our sensitivity to the experiences of care in the participant's story opens up an interpretation of how greater interdependence (being cared for) is a valued outcome from the normatively ambivalent space where care, choice and medical authority intersect. This is because we find that the preferred patient–doctor relationship is that where the affective (the participant feels her concerns about her on‐going symptoms have been heard) and scientific‐bureaucratic authority (authority to send the participant for further investigations) combine. We can then recognise with the participant how the colleague taking responsibility in caring for her is the outcome that is ‘exactly what one loves’. This contrasts to the GP whose caring for the patient can be assumed, but is not told as part of a collaborative or a shared endeavour that adequately sought out the participant's anxieties – or drew on her cultural or epistemic capital – and so is not valued. Our explorations thus far therefore suggest that stories of agency can be indicative of a need for (greater) patient–doctor interdependence and that the importance of being cared *for* needs to be understood beyond (pejorative) descriptions of patients as passive, disempowered or uninformed.

The boundaries of patient expectations are fluid and need to be contextualised within healthcare's contemporary ambivalences. Throughout the above analysis we have identified an ambivalent space that has been described as being treated *in the right way*. This was often alluded to in the interviews and some participants offered detailed and considered accounts of the meaning and process of this experience. As the following participant explains, ideally, good care should include feelings of being involved, while being cared for.

… it's probably the wrong metaphor to use, but being on an escalator or a conveyor, the fact you're in a process; it's a human process so I'm not implying there's no sense of, you know, care or attention to it, but you feel you're part of a process, and you feel all the time that you know what's happening next, and you feel all the time that there is support there if you feel the need of support, whether that is medical support or emotional and sort of psychological support I suppose.

It is interesting to note that the participant is initially reticent in using the conveyor belt as a metaphor. But he seeks to clarify that ‘I'm not implying there's no sense of … care or attention to it’. The normative resonance of the conveyor belt as a metaphor is transformed from representing disinterest and disconnection, to one that helps to express being cared for as a ‘human process’. Indeed, he documents how through continuous medical, emotional and psychological support he feels part of the process of diagnosis from referral, through the investigations, to the results of the histology. As the other participants have also shown us intersections of care, choice and medical authority are (contentiously) negotiated so that experiences of cancer diagnosis are individually contextualised. What we see here is how this relates to cancer diagnosis as a healthcare process characterised by complex oncological knowledge, fractured and bureaucratic healthcare systems, and an emphasis on informed, empowered and responsible patients. Participants’ stories of their cancer diagnosis described doctors who were (or should have been) interested in knowing their situation and who were expected to be active agents in negotiating the various diagnostic processes and epistemic conflicts participants’ faced. In particular, their stories showed how they hoped and sought their doctors to do this in a way that demonstrated a concern for their individual wellbeing and that humanised healthcare's processes.

## Discussion

In this paper we provided a sociological analysis of intersections of care, choice and medical authority in patients’ stories about the patient–doctor relationship during their cancer diagnosis. Approached as a convivial pastoral ambivalence, we found that intersections of care, choice and medical authority that have previously been related to the emancipation of patients from paternalistic health care, were drawn upon by participants to shape stories of interdependency in the relationship between patient and doctor. We found that participants distinguished between a wish for the doctor to engage with them interpersonally in the right way, and the expectation that the interpretation and contextualisation of the medical and diagnostic information needed the doctor's expertise. The expectation that the patient should be an autonomous, rational decision‐maker became problematised by their desire to be guided in their decision‐making or even to give some part of themselves over to the care of the other. As one participant noted when the relationship was going well, ‘you put yourself into the hands of the experts’.

The resilience of this expectation to be cared for, if viewed through a dichotomised patient–doctor relationship, might be understandable as a loss of power and a return to a non‐modern relationship (Buetow *et al*. 2009). However, by approaching emancipatory discourses (such as shared decision‐making, patient centred care and patient empowerment) through the intersections of care, choice and medical authority we were able to emphasise the relational, interpersonal, contextual character of participants’ stories and be sensitive to the limitation of dichotomised framings of the patient–doctor relationship. In this way our analysis of convivial‐pastoral epistemologies shows how participants were finding ways to emplot agency within their stories so that the moral and fiduciary obligations and responsibilities for care could (again) be understood to be shared with their doctor.

This has implications for those who may see sharing and collaborative decision‐making (pejoratively) as a soft or constrained paternalism (Drolet and White 2012, Vinson 2016). The participants’ stories suggest that what needs to be of analytical concern is not only who has power, but also the ways power is expressed and understood. What our analysis adds is a conceptualisation of how that new ambivalence is maintained within the ‘bidirectional affective entanglements’ (Rose, 2009: 74) of the patient–doctor relationship. The participants in this study expected their clinician to keep them informed, *and* help support them to the right decision. How this was accomplished mattered to patients – doctors where expected to be empathetic, open and considerate of the participant's individual needs. In moments of patient–doctor concordance, conviviality shows us how it is possible that there is an ‘indifference to difference’ (Valluvan 2016: 206) or that unequal relations are not necessarily of concern, and pastoral power suggests why: the telos of the patient–doctor encounter remains attuned to the individual and systemic ways the patient can be cared for. Expectations of good interpersonal skills often meant finding ways to understand what was (and should) happen; it did not absolve the doctor from the responsibility for making decisions. Doctors were expected to attend to the interpersonal considerations of sharing information – be aware of what was too much, or too little; when to direct, and when to be circumspect (Ziebland *et al*. 2018). Getting this ‘nannying’ correct allowed both parties to negotiate individual expertise and interpersonal divisions, so that doctors could care for their patients.

The discursive emphasis of previous analysis has focused on the language and administration of health care and decisions with an instrumental consideration of information quality and a dichotomised ontology of the actors (Buetow *et al*. 2009, Harrison and Wood 2000, Shore and Wright 2015). What this analysis has contributed is an interpretation that allows patients’ expectation of expressive and affective engagement with their doctors to transform the basis upon which we might assess and evaluate the attainment of concordance within that relationship. There remained moments when patients want to be told (or at least strongly guided) what to do and others when patients want to make their own decisions. But the mechanisms through which these are to be attained are not only communicative and rational(ised). What our analysis has allowed us to explore are the ways patients sought to be *affected*, if not directed, by their doctors. Importantly, this needed to be experienced as part of a contextually bounded and relationally ambivalent humanity. To be cared for was not understood as an imposition upon a person's freedom when it was part of a healthcare process in which the patient feels engaged as a human *in the right way*. Moreover, being affected, but not directed, by their doctors was often desired by the patient as a way to lessen the emotional and practical burdens of the process of a cancer diagnosis.

### Limitations of the paper

This analysis is focused on stories of the period leading up to a diagnosis of bowel or lung cancer. This is a specific context and cancer has somewhat unique cultural connotations (Kerr *et al*. 2018). We would therefore expect that analysis of normative boundaries of patient experiences of other diseases might differ. We did not interview people who started on the same path but whose investigations did not lead to a cancer diagnosis. We were also not able to detail how (self‐reported) stage of diagnosis might affect stories of patient–doctor relationship preferences (e.g. Brom *et al*. 2014), as not all participants were able to provide information on their diagnostic stage and we did not have access to participants’ medical records. Similarly, there has been considerable investment in the processes and practices around diagnosis and treatments for cancer – from widespread public awareness campaigns to diagnostic pathways such as 2‐week wait etc – that may differentiate patient experiences of the healthcare system (Andersen 2017, Rubin *et al*. 2015, Ziebland *et al*. 2018). As such stories of patient–doctor relations beyond cancer and its diagnosis are therefore likely to include experiences of different normative expectations.

This exploration of normative expectations within participants’ stories does not address what actually took place in the patient–doctor diagnostic encounters, or attend to the doctors’ stories and expectations of these encounters, and nor the does it directly address the healthcare policies, systems or processes in place at the time. It cannot therefore be a full account of either the convivial practices or pastoral relations we have started to explore (Martin and Waring 2018). What we have sought to do is provide reflections on the normative ambivalences found within the participants’ stories about those encounters. In this way we aimed to further previous insights into how contemporary ideals of care, choice and medical authority – and their corollary discussions of empowerment, decision‐making and responsibility – are drawn upon to both constitute participants’ understandings of their cancer diagnosis, and also locate grounds for their and their doctors’ agency in their stories.

## Conclusion

Patients’ stories of their cancer diagnosis process involve expectations of individualised care and empowered choices, which are contextualised within both the technological‐clinical diagnostic processes and the socio‐political context in which interpersonal relations take place. The normative direction of care, choice and medical authority discourses in health care might lead us to believe that patients have never had better access to information, the capacity to act, and be responsible for themselves. Yet we found that some participants were drawing on these discourses as part of a convivial‐pastoral relationship with their doctors. By focusing on the convivial pastoral dynamic we were able to draw attention to ambivalence in participants’ expectations of their doctors to help negotiate the (im)possibilities of being an empowered, autonomous, responsible, choosing patient in an increasing technological, specialised and complex healthcare system.

## Data Availability

In Denmark the data are available for secondary analysis in conjunction with members of the research group named Comparative Cancer Experiences. In England the participants gave informed consent for data to be copyrighted to the University of Oxford for secondary analysis, broadcasting, publication and teaching. In Sweden the data are available for secondary analysis in conjunction with members of the research group named Comparative Cancer Experiences.

## Notes

1 The authors recognise the potential rupture of using conviviality outside of the studies of race and racism studies and considered developing alternative conceptualisations. However, it was felt this would be both academically inappropriate and an injustice to the inspiration and work of those cited as well as the wider field of cultural studies from which it was drawn.

2 Specific ethical approval details for each country are as follows: England: Research Ethics Service reference 14/NS/1035; Denmark: The Biomedical Research Ethics Committee System Act does not apply to this project, as the project does not implicate the use of human biological materials. Standard ethical protocol according to the American Anthropological Association was followed; Sweden: Regional Ethics Board, Lund, Sweden, reg. no 2014/819.
